# Coal tar pitch extract could induce chromosomal instability of human bronchial epithelial cells mediated by spindle checkpoint-related proteins

**DOI:** 10.18632/oncotarget.17025

**Published:** 2017-04-11

**Authors:** Peng Zhang, Zhitao Li, Na Wang, Guangcai Duan, Wei Wang, Yanming Feng, Yong Zhao, Lixia Wang, Hansong Zhu, Qiao Zhang, Xiaozhuan Liu, Weidong Wu, Yongjun Wu, Wu Yao, Jing Wang, Yiming Wu, Feifei Feng

**Affiliations:** ^1^ College of Public Health, Zhengzhou University, Zhengzhou, Henan, China; ^2^ The Affiliated Cancer Hospital of Zhengzhou University (Henan Cancer Hospital), Zhengzhou, Henan, China; ^3^ Medical College, Henan University of Science and Technology, Luoyang, Henan, China; ^4^ School of Public Health, Xinxiang Medical University, Xinxiang, Henan, China; ^5^ Department of Pulmonary, The First Affiliated Hospital of Zhengzhou University, Zhengzhou, Henan, China

**Keywords:** chromosomal instability, coal tar pitch extract, R band, multiplex fluorescence in situ hybridization (M-FLSH), spindle checkpoint

## Abstract

Coal tar pitch (CTP) is a byproduct of coal tar distillation. The workers working with coal tar or in aluminum smelters, potrooms and carbon plants have the opportunities of exposing to coal tar pitch volatiles. Coal tar pitches from which polycyclic aromatic hydrocarbons (PAHs) originate have been shown to exhibit lung carcinogenicity in humans. Chromosomal instability (CIN) is a mechanism in carcinogenesis, however, whether CIN is involved in coal tar pitch-induced lung cancer remains elusive. In this present study, human bronchial epithelial cells (BEAS-2B) were first exposed to coal tar pitch extracts (CTPE) to induce a malignant transformation model. Then, the occurrence of severe chromosomal changes detected using G band, R band and multiplex fluorescence in situ hybridization (M-FISH) staining were examined. It was shown that more clones of transformed BEAS-2B cells at passage 30 following stimulation with CTPE were formed in the soft agar compared with the vehicle control. Moreover, the expression of the spindle checkpoint-related proteins, mitotic arrest defective 2 (Mad2), budding uninhibited in benzimidazole 1 (Bub1), and anaphase-promoting complex (APC), indicators of abnormal chromosomes and carcinogenesis, reduced in CTPE-treated BEAS-2B cells at Passage 30 compared with the vehicle control using real-time PCR and immunohistochemistry. In summary, exposure of BEAS-2B cells to CTPE may induce chromosomal instability through spindle checkpoint-related proteins.

## INTRODUCTION

Coal tar pitch (CTP) is a byproduct of coal tar distillation. It is usually used as coatings and paint base in roofing and paving, and as a binder in asphalt products. A large number of people working with coal tar or in aluminum smelters, potrooms and carbon plants are exposed to coal tar pitch volatiles (CTPVs), which are mainly composed of polycyclic aromatic hydrocarbons (PAHs) [1].

Coal tar pitch has been classified by the International Agency for Cancer Research (IARC) as lung carcinogen in humans [2]. Canadian studies [3, 4] have revealed that the incidences of lung and stomach cancer, as well as other malignancies, were significantly higher in workers exposed to tars. In China, Liu et al. have reported that the standardized mortality ratio (SMR) of lung cancer increased from 149 in unexposed workers to 430 in the highest exposed workers in carbon plants and an aluminum reduction plant [5]. Many animal studies have also shown that CTP is a selective inducer of lung cancer. CTP administration in diet [6] or i.p. injection [7] can induce lung cancer in rodents. However, the molecular mechanisms underlying coal tar pitch-induced lung carcinogenesis have remained elusive, and novel biomarkers for the diagnosis and new targets for the treatment of early stages of coal tar pitch-induced lung cancer need to be specified.

In 1997, Lengauer and Vogelstein first demonstrated evidence of persistent chromosome missegregation in cancer cell lines, initiating the exploration of the role of chromosomal instability (CIN) in tumorigenesis [8]. Up to 2008, CIN has become a hallmark of human neoplasmas [9], which represents abnormalities in both chromosomal number and structure. A direct consequence of CIN is aneuploidy, and many malignancies have been found to exhibit clonal aneuploidy [10–12]. However, the role of CIN in coal tar pitch-induced lung cancer remains unclear.

One potential mechanisms leading to the generation of CIN is spindle checkpoint defects [13]. Spindle checkpoint, also known as the mitotic checkpoint [14, 15], ensures accurate chromosomal segregation and assignment to daughter cells by monitoring whether the chromosomes are correctly attached to microtubules and the direction and tension of the microtubules [16]. When cells enter mitosis, microtubules generated from opposite spindle poles combine with a kinetochore, forming a bi-orientation. Bi-orientation is necessary for accurate chromosomal segregation. However, it occurs randomly for the microtubule and kinetochore combinations, and sometimes linkage errors occur. If such errors are not corrected and cell cycle progression continues, chromosome missegregation will occur to give rise to cells with aneuploidy [17, 18]. Spindle checkpoint defect is considered to be the main cause of aneuploidy in mammalian cells [9, 15], therefore, we hypothesize that spindle checkpoint inefficiency is involved in to CTPE-induced CIN in BEAS-2B cells and lung tumorigenesis.

In this study, human bronchial epithelial cells (BEAS-2B) were used to establish an *in vitro* model of malignant transformation. Bronchial epithelial cells were selected as the *in vitro* model mainly because most lung cancer originates histologically from this cell type. In the current study, CTP extracts (CTPE) were used to stimulate BEAS-2B to induce a malignant transformation model. Subsequent chromosomal changes were evaluated in the transformed cells using G band, R band and multiplex fluorescence in situ hybridization (M-FISH) staining, and the mechanism of spindle checkpoint defect related to these chromosomal changes were explored.

## RESULTS

### Morphological changes in BEAS-2B cells at passage 10, 20, and 30 in the blank, DMSO and CTPE groups

**Figure 1:**
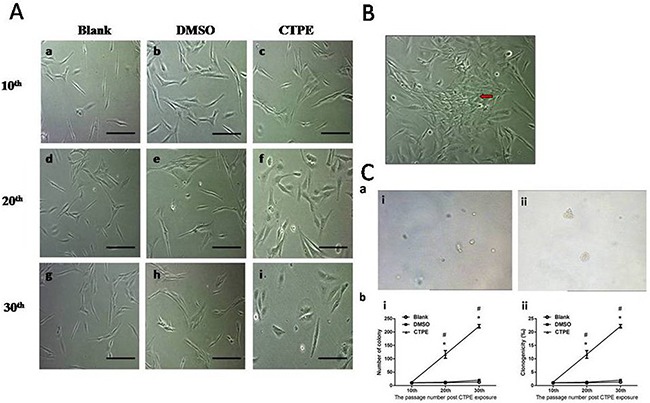
Malignant transformation of BEAS-2B cells at passage 10, 20, and 30 in Blank, DMSO and CTPE groups **(A)** Morphological changes in BEAS-2B cells at passage 10, 20, and 30 in Blank, DMSO and CTPE groups. Scale bar = 50μm. **(a, b, c)** BEAS-2B cells at passage 10 in Blank, DMSO, and CTPE groups. There was no obvious morphological difference of BEAS-2B cells in Blank, DMSO, and CTPE groups at passage 10; **(d, e, f)** BEAS-2B cells at passage 20 in Blank, DMSO, and CTPE groups. BEAS-2B cells in Blank and DMSO groups looked normal; but some BEAS-2B cells stimulated with CTPE were round and/or oval cells increased, and some cells exhibited abundant cytoplasm; **(g, h, i)** BEAS-2B cells at passage 30 in Blank, DMSO, and CTPE groups. There was no obvious change in BEAS-2B cells in Blank and DMSO groups, however, most CTPE-treated BEAS-2B cells were round and oval, some cells were polygonal. **(B)** Irregular growth of CTPE-induced BEAS-2B cells at passage 30. Red arrow shows the cells which lost contact inhibiton and grew in multi-layers. **(C)** Colony formation in soft agar. **(a)** Representatives of colony in soft agar. **(i)** The representative of colonies of BEAS-2B cells in DMSO group at passage 30 in 6-well plate (10×10); **(ii)** The representative of colonies of BEAS-2B cells stimulated with CTPE at passage 30 in 6-well plate (10×10). **(b)** The number of colony and percentage of colonies formation of BEAS-2B cells in soft agar. The number of plating cells on soft agar was 1×10^4^. **(i)** Number of colony; **(ii)** Clonogenicity. *: vs Blank, *P*<0.05; #: vs DMSO, *P*<0.05.

BEAS-2B cells were observed under an inverted microscope, and no obvious morphological differences were observed in the cells from the blank, DMSO, and CTPE groups at passage 10. However at passage 20 and 30, changes were observed in the cells stimulated with CTPE, such as round and/or oval cells and abundant cytoplasm (Figure 1A). CTPE-treated BEAS-2B cells at passage 30 displayed disordered and irregular growth, for example, some cells lose contact inhibition and displayed multi-layer growth (Figure 1B).

### Colony formation of CTPE-induced BEAS-2B cells at passage 20 and 30 in soft agar assay

Figure 1C shows that the number of colonies and percentage of clonogenicity of BEAS-2B cells stimulated with CTPE were not increased at passage 10 but were significantly increased at passage 20 and at passage 30 compared with the other two groups (*P*<0.05).

### Abnormal karyotyping of CTPE-induced BEAS-2B cells by G band staining

**Figure 2:**
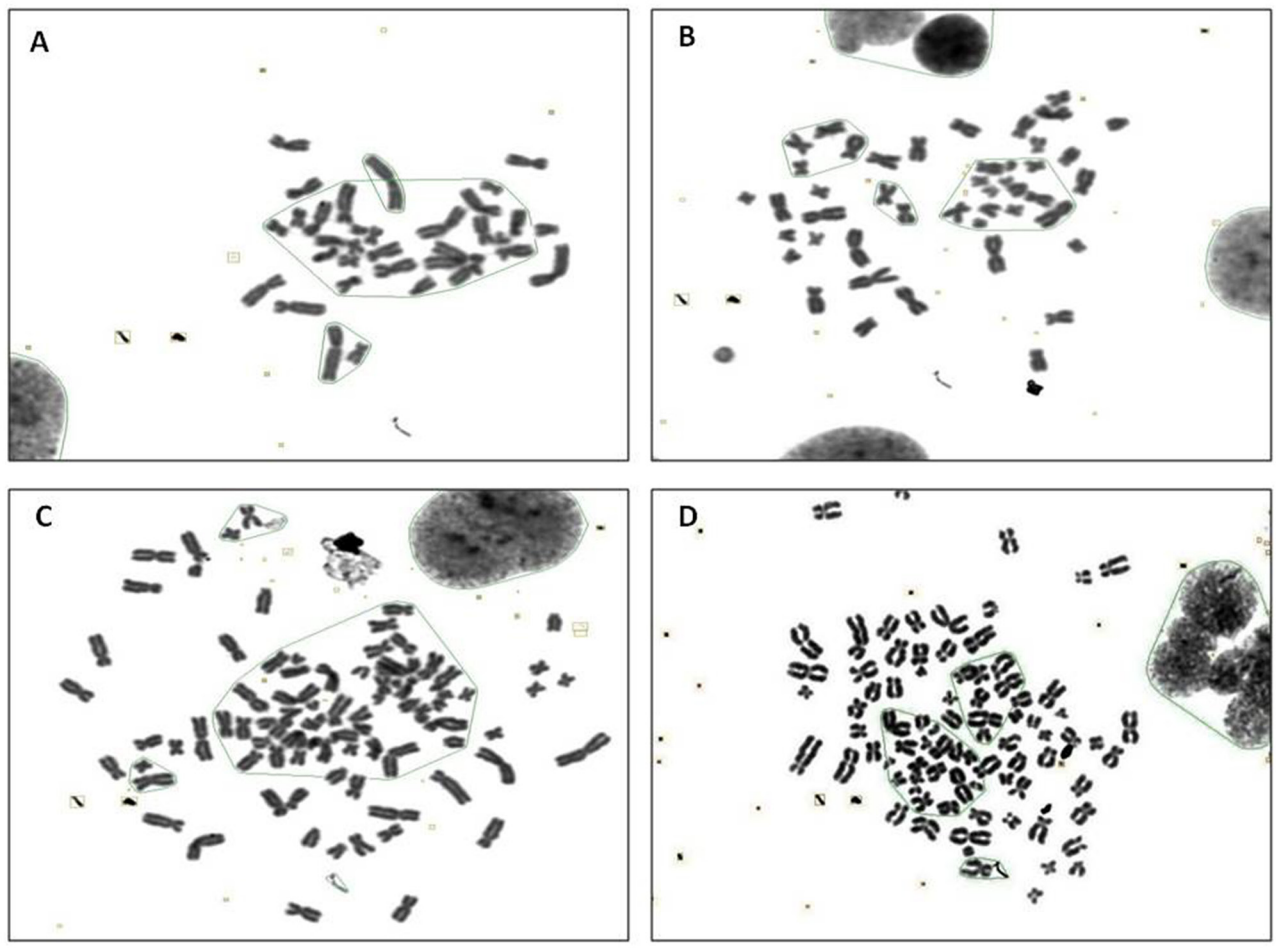
The karyotype representatives of chromosome number changes in BEAS-2B cells by G band staining at passage 30 stimulated with CTPE **(A)** <2n **(B)** 2n **(C)** >3n **(D)** 4n.

**Figure 3:**
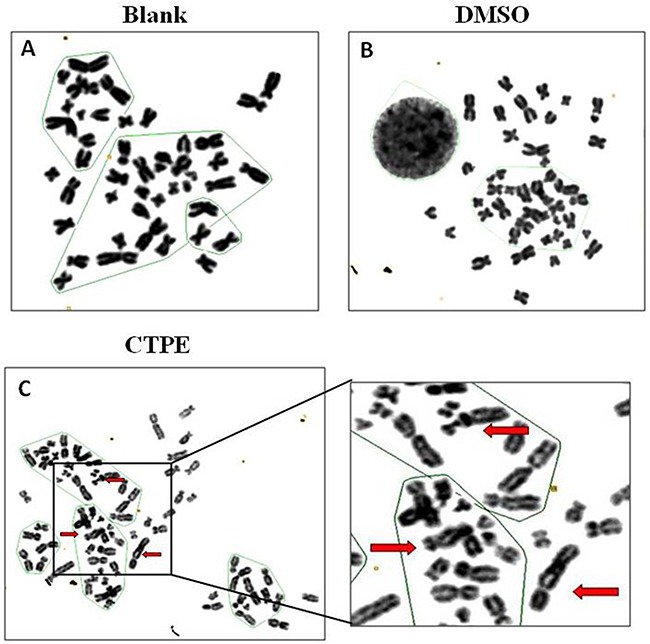
The karyotype representatives of chromosome structure distortion in BEAS-2B cells by G band staining at passage 30 in Blank, DMSO and CTPE groups **(A)** Blank; **(B)** DMSO; **(C)** CTPE. Red arrows: double centromere.

**Figure 4:**
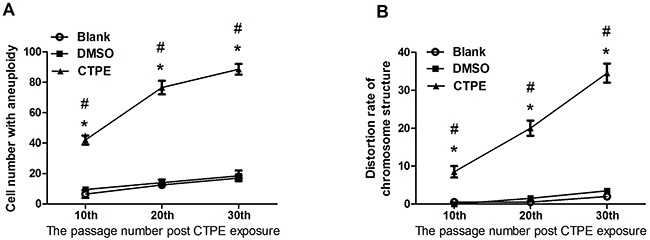
The karyotype of BEAS-2B cells at passage 10, 20, and 30 in Blank, DMSO and CTPE groups **(A)** Cell number with aneuploidy; **(B)** Distortion rate of chromosome structure. *: vs Blank, *P*<0.05; #: vs DMSO, *P*<0.05.

Figure 2 and 3 show representative changes in chromosome numbers and structural distortions in BEAS-2B cells. As shown in Figure 2, aneuploidy includes hypodiploid (<2n) and hyperdiploid (>2n∼≤4n) cells. The red arrow in Figure 3C indicates a double centromere, one kind of chromosome structural distortion. The number of cells with aneuploidy and with chromosome structural distortions among 100 BEAS-2B cells in the CTPE group at passages 10, 20 and 30 were all higher than those in the DMSO group or the blank group (*P*<0.05) (Figure 4).

### Abnormal karyotyping of CTPE-induced BEAS-2B cells at passage 30 by R band staining

**Figure 5:**
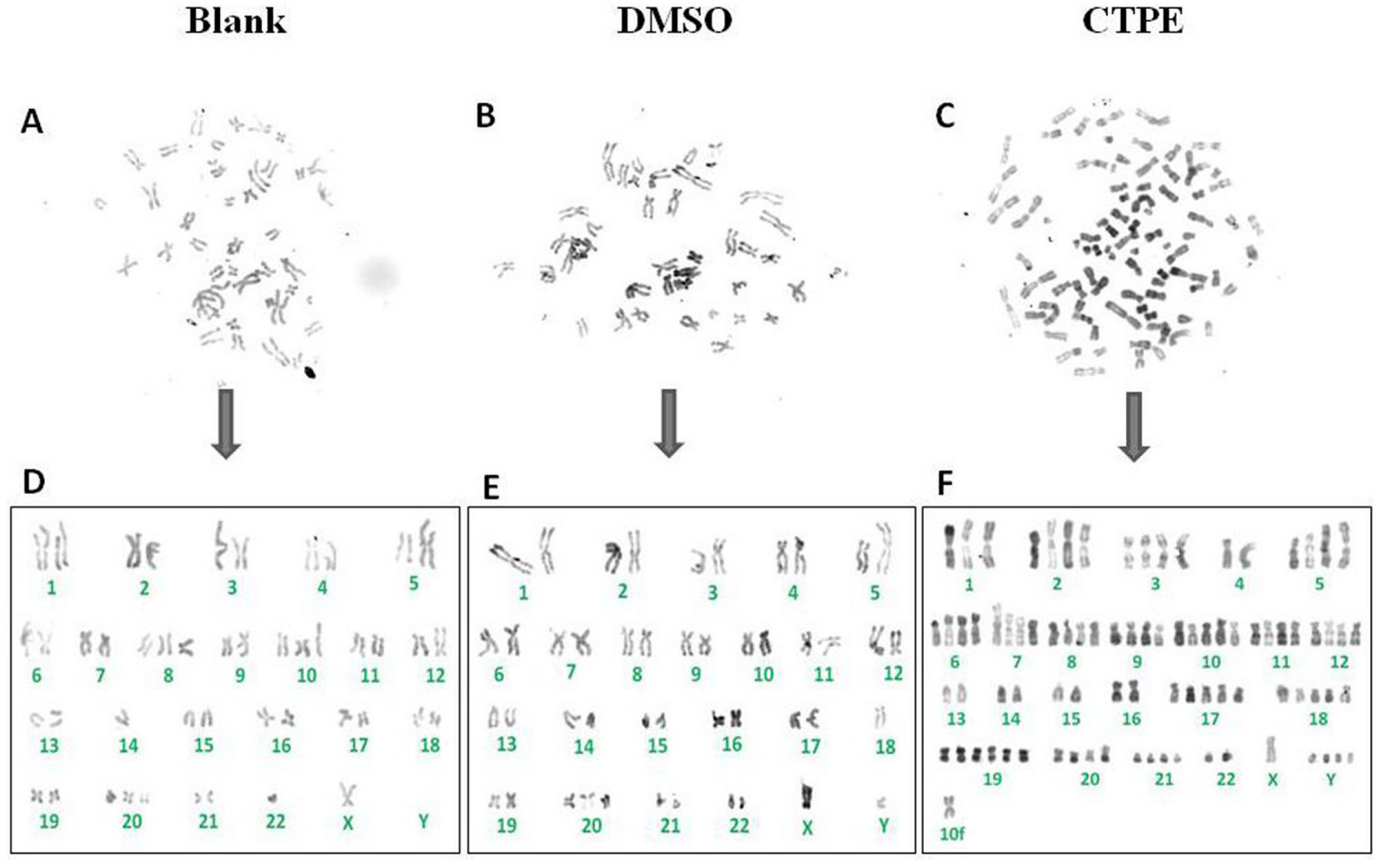
The representatives of the karyotype of BEAS-2B cells by R band staining at passage 30 in Blank, DMSO and CTPE groups **(A, D)** The composite karyotype of BEAS-2B cells by R band staining at passage 30 in Blank group: 2n=46, X, -Y, +8, +10, -14, +20, -22; **(B, E)** The composite karyotype of BEAS-2B cells by R band staining at passage 30 in DMSO group: 2n=46, XY, i(5)(q10), -18, +20; **(C, F)** The composite karyotype of BEAS-2B cells by R band staining at passage 30 in CTPE group: 86 (>3n), XY, +Y, +Y, +Y, +1, +2, +2, +3, +3, +i(5)(q10), +i(5)(q10), +6, +6, +7, +7, +8, +8, +9, +9, +10, +10, +10, +11, +11, +12, +12, +17, +17, +17, +18, +18, +18, +19, +19, +19, +19, +20, +20, +21, +21, +mar.

The R band-stained composite karyotype of BEAS-2B cells at passage 30 in the CTPE group exhibited abnormalities both in chromosome number and structure (Figure 5). In BEAS-2B cells of passage 30 exposed to CTPE, there were 86 chromosomes and abnormal chromosome structures such as the rearrangement i(5)(q10).

### Abnormal karyotyping of of CTPE-induced BEAS-2B cells at passage 30 by M-FISH

**Figure 6:**
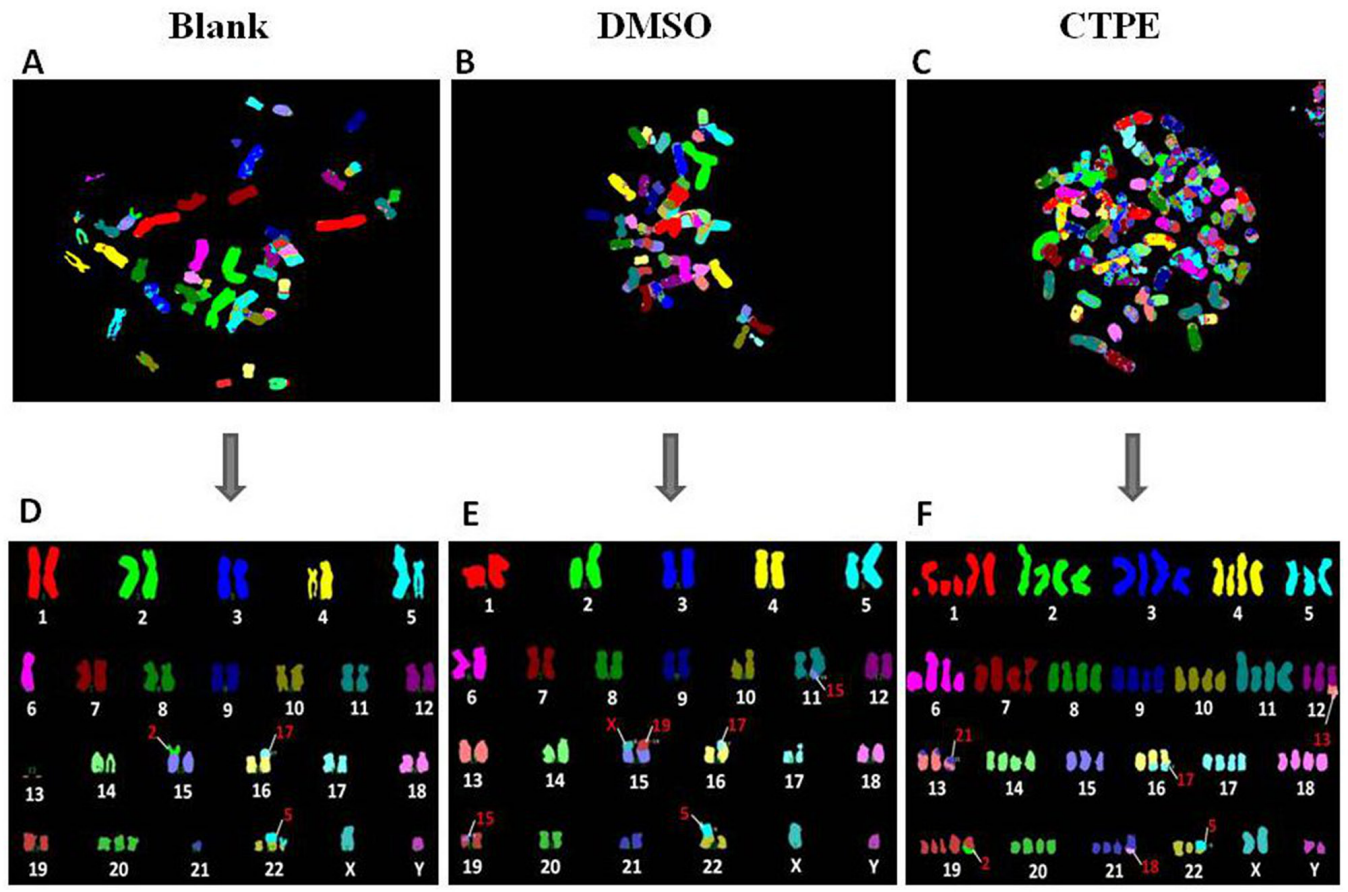
The representatives of the karyotype of BEAS-2B cells by M-FISH at passage 30 in Blank, DMSO and CTPE groups **(A, D)** The composite karyotype of BEAS-2B cells by M-FISH at passage 30 in Blank group: 2n=46, XY, i(5)(q10), -6, -13, -13, t(15;2)(p;p), t(16;17)(p;p), +20, -21, +der(22)t(5;22)(p;p), t(5;22)(p;p) [3]; **(B, E)** The composite karyotype of BEAS-2B cells by M-FISH at passage 30 in DMSO group: 2n=46, XY, i(5)(q10), t(11;15)(q;q), t(15;X)(p;p), t(15;19)(p;p), t(16;17)(p;p), t(19;15)(p;p), t(22;5)(p;p) [4]; **(C, F)** The composite karyotype of BEAS-2B cells by M-FISH at passage 30 in CTPE group: 89(>3n), XXYY, +1, +1q, +1q, +1q, +2, +2, +3, +3, +4, +4, +5, +6q, +6q, +7, +7, +8, +8, +9, +9, +10, +10, +11, +11, +der t(12;13)(q;q), +der t(13;21)(p;p), dup t(13;21)(p;p), +14, +14, +15, +16, dup t(16;17)(q;q), +17, +17, +18, +18, +19, +19, +der t(19;2)(q;q), +20, +20, +21, +der t(21;18)(q;q), +der t(22;5)(p;p), +X, +Y [2].

Figure 6 shows representatives of the composite karyotype of BEAS-2B cells at passage 30 using M-FISH in the blank, DMSO and CTPE groups. Each color represents a chromosome. The data showed a normal number of chromosomes (46) in BEAS-2B cells of passage 30 in the blank and DMSO groups, but in the CTPE group, only 44 chromosomes were observed (<2n). Despite the changes in chromosome structure observed in BEAS-2B cells of passage 30 in all three groups analyzed by M-FISH, the most marked changes were observed in the CTPE group, and these changes were irreversable and could not be repaired.

### mRNA levels of Mad2, Bub1, and APC in BEAS-2B cells at passage 30

**Figure 7:**
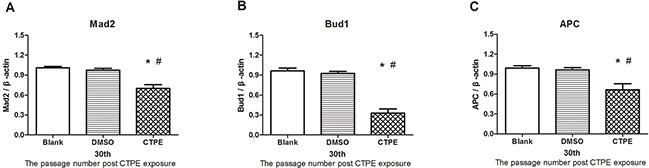
The mRNA expression of Mad2, Bub1 and APC in BEAS-2B cells at passage 30 in Blank, DMSO and CTPE groups The mRNA levels Mad2 **(A)** Bub1 **(B)** and APC **(C)** of CTPE-induced cells were significantly decreased compared with those of BEAS-2B cells in Blank or DMSO group at passage 30. (n=3, *: vs Blank, *P*<0.05; #: vs DMSO, *P*<0.05). Phenotypes were duplicated.

Figure 7 shows significantly decreased mRNA levels of Mad2, Bub1, and APC in CTPE-induced BEAS-2B cells compared with the cells in the blank or DMSO group (all *P*<0.05).

### Protein levels of Mad2, Bub1, and APC in BEAS-2B cells at passage 30

**Figure 8:**
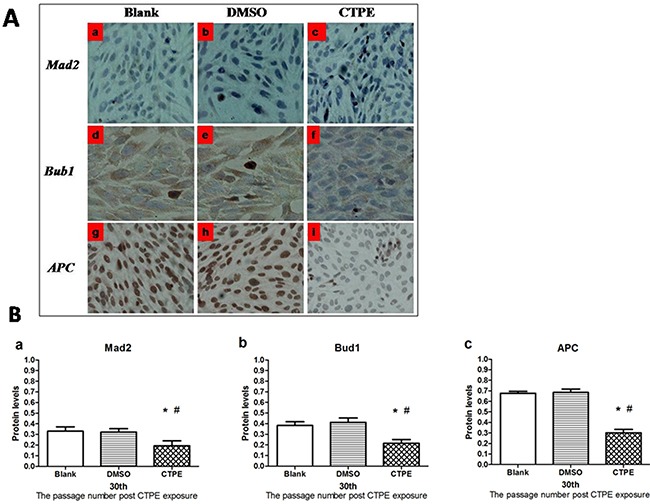
The protein expression of Mad2, Bub1 and APC in BEAS-2B cells at passage 30 in Blank, DMSO and CTPE groups **(A)** Representatives of Mad2, Bub1 and APC protein expression in BEAS-2B cells at passage 30 in Blank, DMSO and CTPE groups (×400). **(a, b, c)** Mad2 protein expression in BEAS-2B cells at passage 30 in Blank, DMSO, and CTPE groups; **(d, e, f)** Bub1 protein expression in BEAS-2B cells at passage 30 in Blank, DMSO, and CTPE groups; **(g, h, i)** APC protein expression in BEAS-2B cells at passage 30 in Blank, DMSO, and CTPE groups. **(B)** The expression levels of Mad2, Bub1 and APC protein in BEAS-2B cells at passage 30 in Blank, DMSO and CTPE groups. The levels Mad2 protein **(a)** Bub1 protein **(b)** and APC protein **(c)** of CTPE-induced cells were significantly lower than those of BEAS-2B cells in Blank or DMSO group at passage 30. (n=3, *: vs Blank, *P*<0.05; #: vs DMSO, *P*<0.05). Phenotypes were duplicated.

The levels of Mad2, Bub1, and APC protein at passage 30 BEAS-2B cells in the three groups were determined using immunohistochemistry, as shown in Figure 8A. Mad2 and Bub1 were detected in the cytoplasm, while APC in the nucleus. The average optical density (AOD) for the protein expression of Mad2, Bub1 and APC in BEAS-2B cells at passage 30 in the different groups is demonstrated in Figure 8B.

The levels of Mad2, Bub1, and APC proteins in BEAS-2B cells were consistent with their mRNA expression levels. As shown in Figure 8B, the levels of Mad2, Bub1, and APC proteins in CTPE-induced BEAS-2B cells at passage 30 were decreased significantly compared with the cells in the blank or DMSO (all *P*<0.05).

## DISCUSSION

The karyotype is the number and appearance of chromosomes in the nucleus of a eukaryotic cell, and can be used to identify early features of cancers and has been associated with tumorigenesis and tumor progression. Chromosome abnormalities include changes in chromosome numbers and structural abnormalities. BEAS-2B cells have stable cytogenetic characteristics. The karyotype of BEAS-2B cells is diploid, and can remain stable during subculturing.

In this study, we detected a small percentage of BEAS-2B cells with aneuploidy in the blank group, and this percentage did not increase significantly with passaging. The morphology of BEAS-2B cells in the blank group from passage 1 to passage 30 looked normal, and the cells in this group were not anchorage-independent. These results imply that it is conditional for cells with aneuploidy to undergo malignant transformation. There are several explanations for the incapability of BEAS-2B cells undergoing malignant transformation in the blank group. First, aneuploidy in the control group may not be of malignance [11, 19]. Second, the cell cycle checkpoints in untreated BEAS-2B cells are normal, which may block the progression from aneuploidy to a malignant karyotype [20]; Third, in some cases aneuploidy can play a role in tumor suppression [11, 20].

In the present study, we examined the morphology of the colonies in soft agar and the chromosomes of BEAS-2B cells stimulated with CTPE, and did not found significant differences in morphology of cells or the number of colonies in soft agar at passage 10 compared with the other two control groups. However, karyotype analysis using G band staining demonstrated that the number of cells with aneuploidy and rate of chromosome structural distortions in CTPE-treated BEAS-2B cells at passage 10 increased significantly compared with the DMSO or blank group, suggesting that chromosomal changes occur during the early stage of malignant transformation, even before the morphological changes take place.

At passage 20, the percentage of CTPE-stimulated BEAS-2B cells with aneuploidy was as high as 80%, indicating the presence of genomic instability, which may promote malignant transformation of stimulated cells [21, 22]. During the early stage, chromosome abnormalities can be eliminated by apoptosis or cell death, but during long-term growth, frequent chromosomal changes may be an important trigger in tumorigenesis [23]. Chromosome segregation displays a high degree of fidelity, which is necessary to maintain genome stability. If there is an abnormality in chromosome segregation, the chromosomes will be allocated to the two sub-cells incorrectly, resulting in chromosomal instability and the generation of aneuploidy cells. In the present study, the number of colonies, clonogenicity, and the percentage of the cells with aneuploidy were increased in CTPE-stimulated BEAS-2B cells at passage 20, indicating that aneuploidy may be involved in tumor cell priming.

When the BEAS-2B cells had reached passage 30, the percentage of cells with aneuploidy was even higher in CTPE-stimulated BEAS-2B cells, resulting in the formation of typical colonies in the soft agar (Figure 1C). The malignant phenotype of the transformed cells, such as polyploid cells, is closely related to tumorigenesis and an important chromosomal feature of cells undergoing malignant transformation. Higher number of abnormal chromosomes is characteristic of malignant cell proliferation, such as higher gene copy number, disordered cell proliferation, and an imbalance in the nuclear cytoplasm ratio. And abnormalities in chromosomal structure, such as abnormal breakage and dicentric chromosomes, may be closely associated with the accelerated proliferation and anchorage-independent growth of malignant cells [22]. Tumorigenicity in nude mice is the most important characteristic of malignant cells, in our previous study of tumor xenograft growth in nude mice, tumors were only observed on the back neck of nude mice transplanted with CTPE-treated BEAS-2B cells of passage 30, but no tumor in blank or DMSO group, or no tumor was formed from passage 10, 20 CTPE-stimulated cells [24]. The results demonstrate that tumor can grow only when there is enough percentage of cells with aneuploidy, showing the importance of CIN in tumor formation.

M-FISH was developed by biomedical researchers in the early 1980s [25] and is used to detect and localize the presence or absence of specific DNA sequences on chromosomes. Even in tumor cells with a highly rearranged karyotype, M-FISH can be used to easily identify subtle changes in chromosomes and complex translocations based on the 24 different colors [26, 27], providing outstanding advantages for tumor karyotype analysis. Figure 6 illustrates representatives of the karyotype of BEAS-2B cells at passage 30 assessed by M-FISH in the blank, DMSO and CTPE groups, respectively. The karyotypes of the BEAS-2B cells at passage 30in the blank and DMSO groups were relatively stable; however, chromosome instability was observed in the CTPE-stimulated BEAS-2B cells, showing a large number of polyploid and sub-diploid karyotypes, and numerous chromosomal displacements and cross-exchanges, as well as missing chromosomes. Different karyotypes were detected in CTPE-treated BEAS-2B cells passage 30, in which no karyotypes were identical, indicating that the chromosomes in these cells were extremely unstable. The results from M-FISH are consistent with the karyotype data obtained using G band and R band staining, which could be used to examine tiny chromosomal changes. An important mechanism that leads to the loss or gain of a few chromosomes during mitosis is impairment of the spindle checkpoint [28]. Minhas and coworkers have reported that there were a greater number of abnormal chromosomes in head and neck squamous carcinoma cells compared with normal control cells. These cells displayed a spindle checkpoint defect, which led to cell CIN [29].

In all cases, changes in gene and/or protein expression of spindle checkpoint proteins, such as Mad2, Bub1 and APC, leads to aneuploidy. Additionally, in most cases, the loss of spindle checkpoint proteins is associated with an increased rate of tumorigenesis, suggesting that aneuploidy promotes tumorigenesis [30]. In this study, we found that gene and protein expression levels of Mad2, Bub1 and APC in malignantly transformed CTPE-treated BEAS-2B cells passage 30 were significantly decreased compared with those in the control groups, indicating that CTPE could reduce the expression of spindle checkpoints-related proteins in abnormal cells, further resulting in aneuploidy.

Mad2 is an essential mitotic checkpoint component that is required for accurate chromosomal segregation during mitosis. A complete loss of Mad2 is lethal, but mice that lack a single copy of Mad2 are viable [31]. Mad2 haploinsufficiency can cause premature anaphase and chromosome instability in mammalian cells, and the incidence of lung tumors has also been found to be significantly increased [31]. It has been demonstrated that Mad2 silencing in gastric cancer cells could contribute to cellular transformation and tumor progression, impacting the microtubules [32]. Bub1 is a serine/threonine protein kinase that targets unattached kinetochores at the onset of mitosis. Bub1 is required for Mad1–Mad2 localization to unattached kinetochores. These complexes (Bub1 and Mad1/2) function to prevent premature anaphase-promoting complex/cyclosome (APC/C) activation by changing the conformation of monomeric Mad2 such that it can efficiently bind to and inhibit the APC/C coactivator Cdc20. The Bub1 hypomorphic mice were highly susceptible to spontaneous tumors, whereas the Bub1 haploinsufficient mice were not. These findings demonstrate that the loss of Bub1 below a critical threshold drives spontaneous tumorigenesis, resulting in chromosome segregation defects [33]. Low levels of Mad2 [34] or Bub1 [33] can induce lung cancer, which is confirmed by this study.

APC/C plays an important role in the “chromosome cycle”, initiating sister chromatid separation. APC/C activation is mediated by Mad1/2 and Bub1. APC is composed of 14 types of subunits, promoting separation of sister chromatids during the transition between metaphase and anaphase during mitosis [35]. In this study, APC expression was decreased and closely related to chromosome missegregation and BEAS-2B cell malignant transformation.

## CONCLUSION

In summary, CTPE exposure could induce alterations in chromosomal numbers and structures in BEAS-2B cells at passage 30, as reflected by G band, R band, and M-FISH staining. These observations were closely associated with cell malignant transformation and lung carcinogenesis. The development of abnormal chromosomes may be mediated by spindle checkpoint genes and proteins such as Mad2, Bub1, and APC. In conclusion, chromosome instability and spindle checkpoint proteins could be used as diagnostic markers and new pharmacological targets for the treatment of coal tar pitch-induced lung cancer.

## MATERIALS AND METHODS

### Preparation of CTPE

CTPE were prepared as described previously [24]. Briefly, moderate-temperature CTP from an Iron and Steel Company was grinded into powder in size of 10∼20μm, and heated at 400°C on the eclectic hot plate in a hood to produce fume. CTP fume was collected on nitrocellulose membrane with a dust sampler. Then the membranes were cut into pieces, dissolved in dichloromethane solution; the solution was filtered through sand core funnel, finally, the filtered supernatant was volatilized in a 45°C drying oven, dimethyl sulfoxide (DMSO) was added to make a final stock concentration of 2 mg/mL.

### Cell line, cell culture and CTPE treatment

The human bronchial epithelial cell line (BEAS-2B) used herein has been described previously [24]. This cell line was derived by transforming human bronchial epithelial cells with an adenovirus 12-simian virus 40 construct [36].

The cell line was cultured in RPMI-1640 medium with 10% (v:v) fetal bovine serum (FBS), 100 IU/mL of penicillin and 100 mg/mL of streptomycin. All cells were cultured at 37°C in a 5% CO_2_ incubator.

BEAS-2B cells were treated with 2.4 mg/mL CTPE (30% of the IC_50_ of 8.11 mg/mL [24]) for 72 h as follows: BEAS-2B cells grown to 70%–80% confluence were treated with CTPE solution for 24 h. After removal of CTPE, the cells were washed with cold PBS and passaged using trypsin-EDTA; the same procedure was repeated two more times for a total of 72 h. DMSO was served as the vehicle control. The medium was used as the blank control. For simplification, the first passage following CTPE first treatment was denoted as passage 0 [24]. The cells were then sub-cultured until passage 30.

### Morphological examination

Coverslips were placed in 6-well plates. BEAS-2B cells at passages 10, 20, and 30 in the blank, DMSO and CTPE groups were placed on coverslips in 6-well plates. Upon the cells 60% confluence, the morphological alterations in the cells of the different passages were observed in the three groups using microscopy (Olympus, Tokyo, Japan).

### Colony formation in soft agar

In a 6-well plate, 1×10^4^ passage 10, 20, and 30 BEAS-2B cells in the blank, DMSO, and CTPE groups were plated in the upper layer (0.7% agar) of the two-layer agar (0.7% and 1.2%). When the agar was solidified, 2 mL of medium was added to each well. The plate was incubated at 37°C in a humidified chamber with 5% CO_2_ for 3 weeks. The medium was changed every 5 days. After 3 weeks, the number of colonies (a colony consisting of more than 50 cells) was counted, and the clonogenicity (‰) was evaluated according to the formulation: the clone number/inoculated cell number × 1000 ‰.

#### Preparation of chromosomes from BEAS-2B cells

BEAS-2B cells at different passages and in three groups in logarithmic phase were treated with 0.04 μg/mL colcemid for 3 h at 37°C to arrest the cells in metaphase. The cells were trypsinized and then centrifuged for 10 min at 1000 rpm, and the cell pellets were resuspended in 37°C KCl hypotonic solution and incubated for 40 min. The swollen cells were pelleted and resuspended in 8 mL of Carnoy's fixation solution (3:1= methanol: glacial acetic acid) at room temperature for 2 h. The cell suspension was centrifuged and washed twice in fixation solution. After the last centrifugation, the cells were resuspended in 2 mL of freshly prepared fixation solution.

### Karyotyping

Karyotype of cells usually can be detected using G band, R band, and M-FISH.

#### G band

G band is named becausethe chromosomes are stained with Giemsa, the most commonly used stain in human cytogenetic analysis and preferentially stains adenine and thymine-rich (AT-rich) regions.

***Procedure***: Slides of chromosomes from the BEAS-2B cells described previously were stained with Giemsa solution (pH 6.8) for 30 min, washed with tap water for 5 seconds, and air-dried. One hundred cells in metaphase were examined for karyotyping (chromosome number abnormality and chromosome structure abnormality).

#### R band

R band is also named reverse band because the R band pattern is essentially the reverse of the G band pattern. To discern the GC-rich R bands, the AT-rich regions are selectively denatured with heat, leaving the GC-rich regions intact. R-band is helpful for analyzing the structure of chromosome ends, which are usually lightly stained by G-band.

***Procedure***: The slides of the BEAS-2B cell chromosomes were placed in a 37°C incubator for 5∼7 d, soaked in PBS (pH 6.8) for 5∼10 min, placed in Earle's balanced salt solution (Thermo Fisher, MA, USA) for 24 h, washed twice with distilled water, and air-dried. The slides were stained with 5% Giemsa solution for 30 min, washed with tap water for 5 seconds, and air-dried. The chromosomes were mounted with neutral gum, and R banding was observed using a high-power microscope. Specific karyotyping of R banding was photographed and analyzed using CytoVision software (Applied Imaging, MI, USA) based on normal human karyotyping. Abnormal karyotypes were organized and marked according to the International System for Human Cytogenetic Nomenclature (ISCN) [37].

#### M-FISH

Multicolor fluorescence in situ hybridization (Multicolor-FISH) is a cytogenetic technique, which utilizes 24 fluorescent probes that can bind only to those parts of the chromosome with a high degree of sequence complementarity.

***Procedure***: The slides of the BEAS-2B cell chromosomes were placed in a 37°C incubator for 5∼7 d, and then the Multicolor FISH kit was applied (Cambio company, Cambridge, UK). The slides were pre-warmed to 45°C in a water bath for at least 30 min, placed in 1x SSC, washed, and then 125 μL of Working Reagent A was added to the slide. The slide was then immediately covered with Parafilm and incubated in a humidified chamber for 15-20 min at 37°C. The Parafilm was removed, and the slide was washed 3 times for 4 min each in a detergent wash solution at room temperature by emptying and refilling the Coplin jar. The slides were air-dried, mounted with 50 μL of DAPI II^®^, and sealed with coverslips. The slides were then viewed using specific epifluorescence filters that were specific for Cy3, Cy3.5, Cy5, Cy5.5, FITC and DAPI II^®^, and the images were captured using a BX61 microscope with a JAI CVM4+ camera and analyzed using CytoVision software (Applied Imaging, MI, USA).

### Real-time PCR

Total RNA was isolated from BEAS-2B cells using the RNAeasy kit (Invitrogen, Carlsbad, CA, USA) according to the manufacturer's protocol. The samples were treated with DNase to remove DNA contamination (Ambion Inc., Austin, TX, USA). RNA samples were then reverse-transcribed into cDNA using SuperScript II RT (Invitrogen Corp., Carlsbad, CA, USA) according to the manufacturer's instructions. mRNA transcription of mitotic arrest defective 2 (Mad2), budding uninhibited in benzimidazole 1 (Bub1), and anaphase-promoting complex (APC), was assessed using the Mx3000P QPCR System (Stratagene, California, USA) with the SYBR^®^ Premix Ex Taq™ (Tli RNaseH Plus) kit (TaKaRa, Tokyo, Japan) and gene-specific primers (Sangon Biotech, Shanghai, China). All values were normalized to the transcription of the housekeeping gene β-actin. The primer sequences for the tested genes and β-actin are listed in Table 1.

**Table 1:** Primers used for RT-PCR

Target gene	Forward primer (5′ to 3′)	Reverse primer (5′ to 3′)	Source
Mad2	GAGTTCTTCTCATTCGGCATCA	CCAATCTTTCAGTTGTTCCACC	PrimerBank
Bub1	TGCTGCACAACTTGCGTCTAC	TCAACGCCCAACTCTGCCT	PrimerBank
APC	CCAACAAGGCTACGCTATGC	ATCTGCTCGCCAAGACAAAT	PrimerBank
β-actin	ATCATGTTTGAGACCTTCAACA	CATCTCTTGCTCGAAGTCCA	PrimerBank

### Immunohistochemistry

The expression of Mad2, Bub1 and APC proteins was assessed by immunohistochemistry. Coverslips were placed in 6-well culture plates. BEAS-2B cells at passage 30 in logarithmic growth phase in each group were collected as a single cell suspension, and the concentration of cells was adjusted to 1×10^6^ cells/mL. The cells were plated in 6-well plates at 1×10^5^ cells/well. The SP method was used to stain the cells on the coverslips for immunohistochemistry after they had reached 80% confluency. Mad2, Bub1 and APC antibodies were purchased from Santa Cruz company, and PBS served as the negative control. The images were recorded with a camera, and the average optical density (AOD) for the protein expression was analyzed using Image-Pro Plus 6.0 software.

### Statistical analysis

The data were processed and analyzed using SPSS12.0 software (SPSS, Chicago, IL, US) and expressed as mean ± standard deviation (x¯ ±s). Differences among groups were examined for statistical significance using one-way analysis of variance, and differences between two groups were examined using LSD test. All of the statistical tests were two-sided and conducted at statistical significance level of P equals 0.05.
